# Supplementary material for: Single-cell new RNA sequencing reveals principles of transcription at the resolution of individual bursts
Source: Nat Cell Biol. 2024 Aug 28;26(10):1725–33. doi: 10.1038/s41556-024-01486-9 (PMC11469958; doi:10.1038/s41556-024-01486-9)
Supplement: Supplementary file 1 — Supplementary Note 1. [file 41556_2024_1486_MOESM1_ESM.pdf]

nature cell biology

Supplementary information https://doi.org/10.1038/s41556-024-01486-9

Single-cell new RNA sequencing reveals principles of transcription at the resolution of individual bursts

In the format provided by the authors and unedited

Supplementary Information

for

Single-cell new RNA sequencing reveals principles of transcription at the resolution of individual bursts

Daniel Ramsköld*, Gert-Jan Hendriks*, Anton J.M. Larsson*, Juliane V. Mayr,
Christoph Ziegenhain, Michael Hagemann-Jensen, Leonard Hartmanis, Rickard Sandberg
Department of Cell and Molecular Biology, Karolinska Institute, Stockholm, Sweden
**equal contribution*
Correspondence to: Rickard Sandberg (Rickard.Sandberg@ki.se)

List of content:

Supplemental Note 1.

Supplementary Note 1: Deriving the probability distribution for the transient period of the random telegraph model

The purpose of this supplemental note is to describe each step in deriving the probability distribution of the telegraph model during in the transient period, i.e. the probability of observing n RNAs t time units after starting the markov process. Deriving this distribution is of course motivated by the development of NASC-seq2, which allow us to count the number of RNAs produced during the 4sU labelling period. While this probability distribution and generating function has previously been described ([1][2][3]), this note details its derivation and is correct.

We begin with the probability generating function

$$\begin{aligned} G(z,t) = M(-k_{on}, 1-k_{on}-k_{off}, k_{syn}e^{-t}(1-z)) \\ \times M(k_{on}, k_{on}+k_{off}, -k_{syn}(1-z)) \\ -\frac{k_{on}k_{syn}e^{-(k_{on}+k_{off})t}}{(k_{on}+k_{off})(1-k_{on}-k_{off})}(1-z) \\ \times M(k_{off}, 1+k_{on}+k_{off}, k_{syn}e^{-t}(1-z)) \\ \times M(1-k_{off}, 2-k_{on}-k_{off}, -k_{syn}(1-z)) \end{aligned}$$

Where M is Kummer's (confluent hypergeometric) function.

To obtain the probability of observing n counts at time t, we compute the nth derivative of $G(z,t)$ with respect to z, evaluated at $z=0$,

$$P(n,t) = \frac{1}{n!}\frac{\partial^n G}{\partial z^n}|_{z=0}$$

Using the general Leibniz rule

$$(fg)^{(n)} = \sum_{r=0}^{n} \binom{n}{r} f^{(n-k)} g^{(k)}$$

where f and g are n-times differentiable functions, $\binom{n}{r} = \frac{n!}{k!(n-k)!}$ is the binomial coefficient and $f^{(k)}$ denotes the kth derivative of f, we can differentiate the three

terms separately. We know that

$$\frac{\partial M}{\partial c} = \frac{aM(a+1, b+1, c)}{b}$$

for arbitrary parameters a, b, c. Since z is always in the third parameter, this knowledge is enough to fully differentiate the whole function. For example the first M function will differentiate as follows

$$\frac{\partial M(-k_{on}, 1-k_{on}-k_{off}, k_{syn}e^{-t}(1-z))}{\partial z} = \frac{-k_{on}k_{syn}e^{-t}}{1-k_{on}-k_{off}} M(-k_{on}+1, 1-k_{on}-k_{off}+1, k_{syn}e^{-t}(1-z))$$

with the nth derivative being

$$\frac{\partial^n M(-k_{on}, 1-k_{on}-k_{off}, k_{syn}e^{-t}(1-z))}{\partial z^n} = \frac{(-k_{on})(-k_{on}+1)...(-k_{on}+n-1)k_{syn}^n e^{-nt}}{(1-k_{on}-k_{off})(1-k_{on}-k_{off}+1)...(1-k_{on}-k_{off}+n-1)} \times M(-k_{on}+n, 1-k_{on}-k_{off}+n, k_{syn}e^{-t}(1-z)) = \frac{(-k_{on})_n k_{syn}^n e^{-nt}}{(1-k_{on}-k_{off})_n} M(-k_{on}+n, 1-k_{on}-k_{off}+n, k_{syn}e^{-t}(1-z))$$

where $(a)_m = a(a+1)...(a+m-1)$.

The differentiation procedure is essentially the same for all other functions. Ultimately, in combination with the general Liebniz rule we obtain the following distribution

$$
\begin{aligned}
&P(n,t) = \\
&\frac{1}{n!}\frac{\partial^n G}{\partial z^n}|_{z=0} = \\
&\frac{k_{syn}^n}{n!}\sum_{r=0}^{n}\binom{n}{r}\frac{(-1)^r(-k_{on})_r(k_{on})_{n-r}e^{-rt}}{(1-k_{on}-k_{off})_r(k_{on}+k_{off})_{n-r}} \\
&\times M(-k_{on}+r, 1-k_{on}-k_{off}+r, k_{syn}e^{-t}) \\
&\times M(k_{on}+n-r, k_{on}+k_{off}+n-r, -k_{syn}) \\
&+\frac{k_{on}k_{syn}^{n+1}e^{-(k_{on}+k_{off})t}}{(k_{on}+k_{off})(1-k_{on}-k_{off})n!} \\
&\times\sum_{r=0}^{n}\binom{n}{r}\frac{(-1)^r(k_{off})_r(1-k_{off})_{n-r}e^{-rt}}{(1+k_{on}+k_{off})_r(2-k_{on}-k_{off})_{n-r}} \\
&\times M(k_{off}+r, 1+k_{on}+k_{off}+r, k_{syn}e^{-t}) \\
&\times M(1-k_{off}+n-r, 2-k_{on}-k_{off}+n-r, -k_{syn}) \\
&-\frac{k_{on}k_{syn}^{n}e^{-(k_{on}+k_{off})t}}{(k_{on}+k_{off})(1-k_{on}-k_{off})(n-1)!} \\
&\times\sum_{r=0}^{n-1}\binom{n-1}{r}\frac{(-1)^r(k_{off})_r(1-k_{off})_{n-1-r}e^{-rt}}{(1+k_{on}+k_{off})_r(2-k_{on}-k_{off})_{n-1-r}} \\
&\times M(k_{off}+r, 1+k_{on}+k_{off}+r, k_{syn}e^{-t}) \\
&\times M(-k_{off}+n-r, 1-k_{on}-k_{off}+n-r, -k_{syn})
\end{aligned}
$$

By letting $t \to \infty$, we recover the well-known steady state distribution

$$P(n) = \frac{k_{syn}^n}{n!}\frac{(k_{on})_n}{(k_{on}+k_{off})_n}M(k_{on}+n, k_{on}+k_{off}+n, -k_{syn}).$$

References

1. Peccoud, J. & Ycart, B. Markovian Modeling of Gene-Product Synthesis. en. *Theoretical Population Biology* **48,** 222–234. ISSN: 00405809. `https://linkinghub.elsevier.com/retrieve/pii/S0040580985710271` (2022) (Oct. 1995).
2. Iyer-Biswas, S., Hayot, F. & Jayaprakash, C. Stochasticity of gene products from transcriptional pulsing. en. *Physical Review E* **79,** 031911. ISSN: 1539-3755, 1550-2376. `https://link.aps.org/doi/10.1103/PhysRevE.79.031911` (2022) (Mar. 2009).

3. Dattani, J. & Barahona, M. Stochastic models of gene transcription with upstream drives: exact solution and sample path characterization. en. *Journal of The Royal Society Interface* **14,** 20160833. ISSN: 1742-5689, 1742-5662. https://royalsocietypublishing.org/doi/10.1098/rsif.2016.0833 (2022) (Jan. 2017).
