# Supplementary material for: Single-cell new RNA sequencing reveals principles of transcription at the resolution of individual bursts
Source: Nat Cell Biol. 2024 Aug 28;26(10):1725–33. doi: 10.1038/s41556-024-01486-9 (PMC11469958; doi:10.1038/s41556-024-01486-9)
Supplement: Supplementary file 2 — Reporting Summary [file 41556_2024_1486_MOESM2_ESM.pdf]

nature portfolio

Corresponding author(s): Rickard Sandberg

Last updated by author(s): May 27, 2024

Reporting Summary

Nature Portfolio wishes to improve the reproducibility of the work that we publish. This form provides structure for consistency and transparency in reporting. For further information on Nature Portfolio policies, see our Editorial Policies and the Editorial Policy Checklist.

Statistics

For all statistical analyses, confirm that the following items are present in the figure legend, table legend, main text, or Methods section.

n/a	Confirmed	
☐	☒	The exact sample size (*n*) for each experimental group/condition, given as a discrete number and unit of measurement
☒	☐	A statement on whether measurements were taken from distinct samples or whether the same sample was measured repeatedly
☐	☒	The statistical test(s) used AND whether they are one- or two-sided<br>*Only common tests should be described solely by name; describe more complex techniques in the Methods section.*
☐	☒	A description of all covariates tested
☐	☒	A description of any assumptions or corrections, such as tests of normality and adjustment for multiple comparisons
☐	☒	A full description of the statistical parameters including central tendency (e.g. means) or other basic estimates (e.g. regression coefficient) AND variation (e.g. standard deviation) or associated estimates of uncertainty (e.g. confidence intervals)
☐	☒	For null hypothesis testing, the test statistic (e.g. *F*, *t*, *r*) with confidence intervals, effect sizes, degrees of freedom and *P* value noted<br>*Give P values as exact values whenever suitable.*
☒	☐	For Bayesian analysis, information on the choice of priors and Markov chain Monte Carlo settings
☒	☐	For hierarchical and complex designs, identification of the appropriate level for tests and full reporting of outcomes
☐	☒	Estimates of effect sizes (e.g. Cohen's *d*, Pearson's *r*), indicating how they were calculated

Our web collection on statistics for biologists contains articles on many of the points above.

Software and code

Policy information about availability of computer code

Data collection	No software was used for data collection.
Data analysis	Analysis of single-cell RNA-seq data was performed as described in detail in the methods. The procrassing of Fastq files were carried out using zUMIs (v. 2.9.7), and aligned to the reference genome using STAR (v. 2.7.1 for human hg38 and v. 2.7.3a for mouse mm39 respectively). Analyses of 4sU base conversions in aligned sequenced data from NASC-seq2 were carried using custom Python code (available at github: https://github.com/sandberg-lab/NASC-seq2), as well as and further downstream analyses.

For manuscripts utilizing custom algorithms or software that are central to the research but not yet described in published literature, software must be made available to editors and reviewers. We strongly encourage code deposition in a community repository (e.g. GitHub). See the Nature Portfolio guidelines for submitting code & software for further information.

Data

Policy information about availability of data

All manuscripts must include a data availability statement. This statement should provide the following information, where applicable:

- Accession codes, unique identifiers, or web links for publicly available datasets
- A description of any restrictions on data availability
- For clinical datasets or third party data, please ensure that the statement adheres to our policy

Raw NASC-seq2 sequencing data (K562 and primary fibroblast cells) have been deposited in ENA (accession id: PRJEB60799) and source data has been deposited in

Zenodo (https://doi.org/10.5281/zenodo.11350119). Kinetic estimates and count tables are available on github (https://github.com/sandberg-lab/NASC-seq2). We downloaded genome sequences from UCSC Genome Browser (mouse: GRCm39/mm39 and human:GRCh38/hg38) and Gencode gene annotations (human ENSEMBL GRCh38.95 and mouse GRCm39.vM29).

Research involving human participants, their data, or biological material

Policy information about studies with human participants or human data. See also policy information about sex, gender (identity/presentation), and sexual orientation and race, ethnicity and racism.

Reporting on sex and gender	Not applicable
Reporting on race, ethnicity, or other socially relevant groupings	Not applicable
Population characteristics	Not applicable
Recruitment	Not applicable
Ethics oversight	Not applicable

Note that full information on the approval of the study protocol must also be provided in the manuscript.

Field-specific reporting

Please select the one below that is the best fit for your research. If you are not sure, read the appropriate sections before making your selection.

☒ Life sciences ☐ Behavioural & social sciences ☐ Ecological, evolutionary & environmental sciences

For a reference copy of the document with all sections, see nature.com/documents/nr-reporting-summary-flat.pdf

Life sciences study design

All studies must disclose on these points even when the disclosure is negative.

Sample size	Sample sizes were not predetermined using statistical analysis, however the power to infer new and old transcripts based on length of RNA sequencing was determined as shown in the study.
Data exclusions	Single-cell RNA-seq data were filtered according to established criteria. Cutoffs are listed where appropriate.
Replication	All experiments were performed across hundreds to tens of thousands of individual cells, and kinetic analyses from subsets of cells showed in general good agreement.
Randomization	Cells were randomly sorted into microwell plates using FACS, 4sU and no-4sU control cells, present on each plate when possible.
Blinding	Investigators were not blinded to groups of samples, since we only analyzed one type of mouse fibroblasts and one group of human K562 cells.

Reporting for specific materials, systems and methods

We require information from authors about some types of materials, experimental systems and methods used in many studies. Here, indicate whether each material, system or method listed is relevant to your study. If you are not sure if a list item applies to your research, read the appropriate section before selecting a response.

Materials & experimental systems

n/a	Involved in the study	
☒	☐	Antibodies
☐	☒	Eukaryotic cell lines
☒	☐	Palaeontology and archaeology
☐	☒	Animals and other organisms
☒	☐	Clinical data
☒	☐	Dual use research of concern
☒	☐	Plants

Methods

n/a	Involved in the study	
☒	☐	ChIP-seq
☒	☐	Flow cytometry
☒	☐	MRI-based neuroimaging

Eukaryotic cell lines

Policy information about cell lines and Sex and Gender in Research

Cell line source(s)	K562 cells from DSMZ (ACC-10).
Authentication	K562 cells were authenticated at the DSMZ Identification Service according to standards for STR profiling (ASN-0002).
Mycoplasma contamination	Cells were tested for Mycoplasma and were confirmed negative (MycoAlert, Lonza).
Commonly misidentified lines (See ICLAC register)	No commonly misidentified lines were used in this study.

Animals and other research organisms

Policy information about studies involving animals; ARRIVE guidelines recommended for reporting animal research, and Sex and Gender in Research

Laboratory animals	Primary mouse fibroblasts were obtained from the tail of a 5 month old female CAST/EiJ x C57BL/6J mouse, as described in the metods section. Animals were housed in standard housing conditions (ambient temperature of 20-22°C and humidity of 40-60%) with 12:12-hour light:dark cycles with food and water ad libitum. All experimental procedures were approved by the Stockholms Norra Djurförsöksetiska Nämnd.
Wild animals	No wild animals were used in this study.
Reporting on sex	Large experiment on primary mouse fibroblasts were performed on a female (in order to have X-chromosome inactivation as an allellic control), the conclusions on general transcriptional bursting and co-bursting should not differ substantially between sexes.
Field-collected samples	No field-collected samples were used in this study.
Ethics oversight	Ethical permit numbers N95/15 and 13572-2020 from Jordbruksverket (Sweden).

Note that full information on the approval of the study protocol must also be provided in the manuscript.
